# Selfies, narcissism and oral health a cross - sectional study among adolescents and young adults in Mangaluru - India.

**DOI:** 10.12688/f1000research.153818.2

**Published:** 2024-09-26

**Authors:** Preeti Prabhu, Mithun Pai, Shweta Yellapurkar, Srikant Natarajan, Amit Vasant Mahuli

**Affiliations:** 1 Public health Dentistry, Manipal College Of Dental Sciences, Mangalore, Manipal Academy of Higher Education., Karnataka, Manipal, 576104, India; 2 Department of Oral Pathology and Microbiology, Manipal College of Dental Sciences, Mangalore. Manipal Academy Of Higher Education, Karnataka, Manipal, 576104, India; 3 Public Health Dentistry and Preventative Dentistry, Dental Institute, Rajendra Institute of Medical Sciences, Ranchi, Jharkhand, 834001, India

**Keywords:** Adolescents, Narcissism, Oral health, perceived health, selfie.

## Abstract

**Background:**

Selfies have been defined as “a photograph that one has taken of oneself, and are a continuing growing trend among Millennials. Narcissism refers to the personality trait of an extraordinary self-awareness and adoration. Studies have shown a close relationship between selfie-taking behaviour, narcissism and oral health. Hence, a study was conducted with aim of the influence of selfie-taking behavior and narcissism on oral health.

**Methods:**

An analytical cross-sectional questionnaire study was designed using relevant literature for collating information on sociodemographic characteristics, frequency of dental visits, selfie-taking behavior and perceived oral health. Oral health examination was performed to assess decay and plaque. Narcissism was assessed using The Five-Factor Narcissism Inventory Short Form

**Results:**

The study included a sample of 163 participants. The associations between selfies taken per day and perceived oral health was statistically significant (p value 0.047*). Binary logistic regression models were constructed, which were significant for perceived oral health and grandiose fantasy. Plaque Scores showed significant corelations between indifference, manipulativeness, thrill seeking and age. Step forward binary regression demonstrated a significant odds ratio for grandiose narcissism followed by selfie-taking behavior

**Conclusion:**

This study revealed a correlation between perceived oral health and selfie-taking behaviour. Further studies are required to assess selfie-taking behaviour and oral health.

## Background

Selfies have been defined as “a photograph that one has taken of oneself, typically one taken with a smartphone or webcam and shared via social media” and are a continuing growing trend among Millennials (Generation Y). ^1^ Selfies are now correlated with uploading the same on social media platforms, mainly because of the global saturation of smartphones with cameras and the indispensable integration of this with the presence of Social networking sites (SNS), applications such as Facebook, Twitter, WeChat, Tumblr, Snapchat and Instagram. Selfies, as a new social trend, warrant greater academic consideration, and the intention behind selfie taking is still an enigma in the field of psychology, which warrants further exploration of the intent behind this behavior. Not much is known about selfie addiction in general, and selfies, in particular, have come under increased scrutiny because of their disbursing nature, and experts in the field of psychology and communication are trying to investigate this phenomenon in detail. ^2^ Over the past few years, the triple digital revolution of social networks, the internet, and mobile phones has increased the use and popularity of selfies.

Narcissism refers to the personality trait of an extraordinary self-awareness and adoration often referred as “self-love that shut out everyone else”. ^3^ The word “narcissism” is typically associated with increased self-worth, feelings of grandiosity, dominance, admiration seeking, and privilege (entitlement), which forms a part of narcissism called the grandiose narcissism, but there also is a less prominent trait called Vulnerable narcissism which includes self-absorption, self-consciousness, social insecurity, and defensiveness. Together they revolve on the fundamental idea of self-centered egocentric behavior; they initially appeared as the “two faces” of narcissism and show up as radically distinct personality traits. ^4^

Personality and its developmental aspects among individuals are extending the frontiers in psychology. Studies have shown efficient integration between the Big Five personality traits and various dimensions of SNSs, such as communication patterns and social media language, along with a wealth of vocabulary. ^5^ Additionally, there is a direct association between the Dark Triad and personality traits, as mentioned earlier. Additionally, psychological traits could also be related to selfie posting behavior. Therefore, selfies are no longer considered merely technological artifacts. Instead, it is considered a means of communication and a purposeful symbolic gesture. ^6^

Adolescence is described as a transitional phase between childhood and adulthood, with a biological beginning (puberty) and a social ending. Adolescents are a susceptible group; they are the connect between young and old and are often the population that is routinely under scrutiny because of their diverse physical and psychological traits.

Perceived oral health depends on factors such as well-being, self-satisfaction and, most importantly, oral health habits such as the frequency of tooth brushing, flossing and dental visits. A study conducted on the influence of narcissism on oral health status showed that good/excellent self-rated oral health status was associated with high levels of narcissism. ^7^

The study was envisaged with the aim and objective of assessing correlations between perceived health, perceived oral health, selfie-taking behavior, narcissism and oral health status using a standard questionnaire among a population of college students in Mangaluru, Karnataka.

## Methods

This Analytical Cross-sectional study was carried out in the city of Mangaluru, located on the west coast of Karnataka, India in the district of Dakshina Kannada. The study was conducted from 2019-2021 as part of an oral health screening programme at the college as part of the institutional health screening programme. The students from the Department of Business Studies were the participants in the study. A Convenient sample was obtained and all students from the department who were eligible for study were included in the study.

**Eligibility criteria:** Individuals who declined to give their consent were excluded from the study. The study comprised participants who were present during the study process and could communicate in English as the questionnaire was in English only.

Participants who were receiving psychiatric therapy or who were suffering from generalised anxiety disorders, who mentioned it in the questionnaire were excluded from the study.

**Ethics:** The approval was obtained from the Institutional Ethics Committee. [Protocol No: 18018]. Permission was obtained from the head of the institution of the college. Participants provided written informed consent before answering the questionnaire and undergoing an oral examination. Participants in need of urgent oral care were referred to the institution for oral emergency care.

### Data collection procedure

A Self-administered questionnaire which included demographic information (age, sex, education, family monthly income, previous dental visits and medical history), selfie-taking behaviour, a single question on perceived oral health and perceived general health were include with closed-ended responses. Narcissism was assessed using a short questionnaire version of The Five-Factor Narcissism Inventory Short Form (FFNI-SF). Glover et al. (2012) created the Original Five-Factor Narcissism Inventory (FFNI), a 148-item self-report tool with 15 features meant to evaluate the essential factors of narcissism. ^8^ The FFNI assesses both variants of namely vulnerable and grandiose narcissism. The Five-Factor Narcissism Inventory Short Form (FFNI-SF) was conceived by Sherman et al. (2015), as the original FFNI was extensive and not practical for overall use. The sixty-item FFNI-SF assesses three higher-order qualities (neuroticism, extraversion, and antagonism) as well as fifteen traits that can be combined to evaluate grandiose and vulnerable narcissism. It was also established that the constituents of the FFNI-SF can be valid and reliable. ^9^^–^^11^

### Statistical analysis

The Statistical Package for Social Sciences (SPSS), version 16.0 (SPSS Inc., Chicago IL), was used to analyze the data. We computed the mean and standard deviation for describing the data. The relationships between the five-factor narcissism subscale domain scores and the practice of taking selfies were examined using independent t tests. The chi-square test and Fisher’s exact test were used for comparisons of selfie-taking behaviour and perceived oral health, perceived general health, DMFT score and the plaque index. Pearson product-moment correlation was used to assess the correlation between selfie-taking behavior (Taking more than three selfies per day) and different traits of narcissism, namely, grandiose and vulnerable narcissism, antagonism, extraversion, and neuroticism. Binary logistic regression was used to predict perceived oral health using narcissism, selfie behavior and gender.

## Results

The study included a sample of 163 participants, 159 of whom completed the study, with a response rate of 97%, 96 (60.3%) of whom were males and 63 (39.7%) of whom were females. The sample had a mean age of 18.03 years, with a 4.3-year standard deviation, 71.1% had visited a dentist before this study was conducted. The mean monthly family income of participants was Rs 20810 (SD 7300).

The Pearson chi-square test was used to assess the associations between selfie-taking behaviour, perceived general health, perceived oral health, dental caries experience, and the plaque index. The associations between selfies taken per day and perceived oral health was statistically significant (p value 0.047*), but there was no association with other variables, as described in Table 1.

**Table 1.** Association between selfie behaviour, perceived health, oral health, dental caries and Plaque.

Outcome variable		Selfie behaviour - Absent	Selfie behaviour - Present	X ^2^	p
Perceived general health	Good	13 (8.17%)	76 (47.7%)	0.22	0.63
	Fair	9 (5.66%)	56 (35.2%)		
	Poor	0	5 (3.14%)		
Perceived oral health	Good	12 (7.54%)	101 (63.5%)	6.12	0.047*
	Fair	10 (6.23%)	30 (18.8%)		
	Poor	0	6 (3.77%)		
DMFT index	≤3	17 (10.69%)	109 (68.5%)	0.06	0.806
	>3	5 (3.14%)	28 (17.6%)		
Plaque index	Good	13 (8.17%)	76 (47.7%)	0.847	0.655
	Fair	9 (5.66%)	56 (35.2%)		
	Poor	0	5 (3.14%)		

The Pearson correlation coefficients between total narcissism, grandiose narcissism, vulnerable narcissism, dental caries, perceived oral health, and the plaque index showed a statistically significant positive correlation between perceived oral health and vulnerable narcissism and total narcissism, but there was no statistically significant correlation between grandiose narcissism or dental caries experience and plaque scores. However, a significant negative correlation was observed between grandiose narcissism and the plaque index. The correlations between all the covariables are detailed in Table 2. Further evaluation between selfie behaviour and covariables revealed a positive significant correlation between gender and selfie behaviour.

**Table 2.** Pearson correlation between types of Narcissism, Dental caries, Perceived oral health, Plaque index.

Covariates		Perceived oral health	DMFT	Plaque index	Total Narcissism	Grandiose Narcissism
DMFT	r	0.099	-	-	-	-
	p	0.214	-	-	-	-
Plaque index	r	-0.038	0.175*	-	-	-
	p	0.637	0.028	-	-	-
Total Narcissism	r	0.162*	0.065	-0.112	-	-
	p	0.042*	0.419	0.159	-	-
Grandiose Narcissism	r	0.131	0.064	-0.155*	0.977**	-
	p	0.101	0.427	0.050*	0.00	-
Vulnerable Narcissism	r	0.207	0.051	0.036	0.807**	0.664**
	p	0.009*	0.524	0.655	0.00	0.00

Independent t tests were used to assess differences between selfie-taking behavior and differing traits of narcissism. The test demonstrated no significant association between the various traits of narcissism and selfie behavior, as presented in Table 3.

**Table 3.** Independent t test, selfie behaviour and different traits of narcissism.

	Selfie taking behaviour Absent (n=22)	Selfie taking behaviour (n=137)	t	p
	Mean±SD	Mean±SD		
Acclaim seeking	12.68±3.48	13.52±2.85	-1.238	0.218
Arrogance	11.59±2.36	11.37±2.67	0.374	0.709
Authoritativeness	12.59±2.81	11.86±2.6	1.208	0.229
Distrust	12.55±1.87	12.18±2.18	0.739	0.461
Entitlement	11.77±2.41	11.52±2.63	0.426	0.671
Explottativeness	10.77±2.41	9.79±3.28	1.349	0.179
Grandoise fantasy	12.95±1.79	13.16±2.35	-0.393	0.695
Indifference	12.82±3.39	13.33±2.86	-0.756	0.451
Lack of empathy	11.45±2.28	11.5±2.94	-0.064	0.949
Manupulativeness	11.05±2.42	11.55±2.78	-0.811	0.418
Need admiration	11.68±1.94	11.71±2.26	-0.051	0.959
Reactive anger	12.86±2.95	13.2±3.93	-0.389	0.698
Shame	12.23±3.05	12.69±2.87	-0.691	0.491
Thrill seeking	11.23±2.99	12.16±3.36	-1.225	0.222
Exhibitionist	12.77±3.04	12.49±3.04	0.406	0.685
antagonism	93.27±14.6	93.27±16.45	0.001	0.999
extraversion	51±9.71	51.03±8.4	-0.015	0.988
Neurotisim	36.73±7.36	37.72±5.98	-0.701	0.485
Grandoise Narcissism	131.68±22.41	132.24±22.03	-0.11	0.912
Vulnerable Narcissism	49.32±8.04	49.78±7.88	-0.255	0.799

Binary logistic regression models were constructed for the prediction of perceived oral health, DMFT index, presence or absence of caries and plaque, traits of narcissism, gender, and selfie-taking behaviour. The odds were significant for perceived oral health and grandiose fantasy. Plaque Scores showed significant corelations between indifference, manipulativeness, thrill seeking and age. Multiple charts for binary logistic regressions are collated with only odds ratios, and unstandardised regression rates for better understanding and presented in Table 4. ^21^

Tables 5 and 6 show the results of forward stepwise binary logistic regression for the prediction of poor perceived oral health and plaque indices using the traits of narcissism, gender, and selfie behaviour. The step forward binary regression demonstrated a significant odds ratio for grandiose narcissism followed by selfie-taking behavior as the key variable associated with plaque behaviour.

**Table 5.** Plaque index prediction using all variables using binary logistic regression forward stepwise.

		B	S.E.	Wald	p	Odds Ratio (OR)	95% C.I. OR	
							Lower	Upper
Step 1 ^a^	AGE	-0.920	0.18	24.71	<0.01 ^**^	0.39	0.27	0.57
	Constant	17.15	3.49	24.14	<0.01 ^**^	28194529.54		
Step 2 ^b^	Thrill seeking	-0.13	0.05	5.29	0.021	0.87	0.78	0.98
	AGE	-0.86	0.19	20.50	<0.01 ^**^	0.42	0.29	0.61
	Constant	17.62	3.60	23.84	<0.01 ^**^	45098881.29		
Step 3 ^c^	Indifference	0.16	0.07	4.63	0.031 ^*^	1.18	1.01	1.37
	Thrill seeking	-0.21	0.07	9.02	0.003 ^*^	0.81	0.70	0.93
	AGE	-0.92	0.19	21.87	<0.01 ^**^	0.39	0.27	0.58
	Constant	17.45	3.65	22.84	<0.01 ^**^	37999770.388		

**Table 6.** Forward stepwise binary logistic regression: prediction of Poor Perceived oral health.

		B	S.E.	Wald	p	Odds Ratio	95% C I for ODDS RATIO	
							Lower	Upper
Step 1 ^a^	Grandiose fantasy	0.24	0.08	8.83	0.003	1.280	1.08	1.50
	Constant	-4.19	1.14	13.52	0.000	0.015		
Step 2 ^b^	Grandiose fantasy	0.26	0.08	9.47	0.002	1.299	1.10	1.53
	Selfie behaviour	-0.97	0.48	4.03	0.045	0.376	0.145	0.977
	Constant	-3.58	1.19	9.06	0.003	0.028		

## Discussion

Adolescence is a period of profound physical, psychological, and behavioral transformation during which adolescents form habits and behaviors that they carry into adulthood. In addition, there are more specific risk factors linked to poor dental health at the period. Adolescents constitute a major part of the population, yet there are still limited studies regarding the population, especially in dental settings. ^12^

The main objectives of the study were to evaluate the associations between perceived health, perceived oral health, selfie-taking behavior, narcissism and oral health status in an adolescent population in Karnataka, India. Multiple studies have assessed the association between selfie-taking behaviour and narcissism, ^12^^,^^13^ and studies have also demonstrated the effect of narcissistic personality disorders on oral health. ^4^^,^^7^^,^^14^

The instrument used for this study was a validated version of the short Form of Five-Factor Narcissism Inventory with the following characteristics: a Cronbach’s alpha of 0.8, a split half of 0.8 and a test-retest reliability of 0.71 (p<0.01), as tested by the authors on a population similar to that used in the study. The Five-Factor Narcissism Inventory Short Form (FFNI-SF) was created because the original Five-Factor Narcissism Inventory (FFNI) was too lengthy and unworkable for general usage. The 60-item FFNI-SF assesses the three higher order elements of narcissism anger, extraversion, and neuroticism as well as the same 15 characteristics that can be combined to produce grandiose and vulnerable narcissistic presentations. Sherman et al. ^9^ reported that the sixty items of the FFNI-SF may still be used to measure the original FFNI’s constituents in a valid and reliable manner, boosting the instrument’s efficiency while maintaining its validity and reliability. ^11^ Narcissism has been explored in terms of subtypes rather than as a singular construct. Grandiose (the overt kind) and vulnerable (the covert type) are two of the most discussed forms of pathological narcissism. ^10^

The associations between selfie taking behaviour and perceived general health, perceived oral health, dental experience and plaque scores were significant only between selfie behaviour and perceived oral health. There have been limited in the literature that have examined selfie behaviour in terms of perceived health, perceived oral health and dental status in a population, which is understandable because selfies and oral health were considered unrelated phenomena or because no close associations are observed between them; however, recently, the upsurge in social media and its close association with selfie behaviour have led investigators to scrutinise this phenomenon on the whole. Studies in South Africa ^15^^,^^16^ have pointed out that dental health, especially perceived dental health, is closely related to selfie behaviour, as people with malocclusion are averted from smiling in selfies; hence, this may explain the association between these two factors. Adolescents lead a carefree life with less importance to health, a healthy lifestyle and well-being; hence, they usually have positive perceptions of health as generally being positive regardless of what they might be going through; hence, there might be no association with perceived health and other factors.

A positive correlation was observed between total narcissism, vulnerable narcissism and perceived oral health. Research has shown that there are two main narcissistic variants, namely, grandiose and vulnerable narcissism. The primary difference between the two variants is self-regulation. Grandiose narcissists typically use overt techniques to control their ego, such as discounting others and enhancing their own worth, whereas vulnerable narcissists typically depend on social acceptance to control their delicate ego. Vulnerable types are more likely to recognise and counteract challenges to their self-image. Hence, there may be a positive correlation between perceived oral health and vulnerable narcissism, and people with high levels of vulnerable narcissism may experience dissatisfaction from their narcissistic demands for approval and achievement. ^17^

They are also more self-vigilant and conscious about self-presentation. Hence, oral health, especially the aesthetic part of the face, is important for these subtypes, which can also be a reason for such correlation. ^18^

Narcissism has a close relationship with oral health parameters. The present study revealed a positive correlation between grandiose fantasy and perceived oral health. Participants with grandiose fantasy ^19^ have closely studied the effects of grandiose fantasy on personality traits and believe that people with grandiose fantasy tend to feel better for themselves. It is also proposed that grandiose fantasising is highly prevalent in both traits of narcissism, hence the reason for a better perception of oral health. ^20^ The traits such as indifference, manipulativeness and thrill seeking was positively related to the plaque index, where higher plaque index scores were observed in subjects with indifference, manipulativeness and thrill seeking. Compared with individuals with other personalities, individuals with narcissistic personalities who are exploring oral health behaviors are less likely to visit dentists for check-ups or for tooth cleaning and scaling. ^7^

### Limitations of the study

The present study has limitations that merit attention. First, as noted above, we are attempting to predict behaviours at a relatively young age the possible relations might need to be explored later in development and might change during further course of life. Future research should investigate these developmental links later in young adulthood when these behaviours become more common. Furthermore, our study design does not allow any causative explanations. The study was done in a single setting and had more male participants, this might affect the overall outcome variables as multiple studies have pointed out the effect of selfie and narcissism to be corelated to females than males.

## Conclusion

This research establishes a significant correlation between the act of taking selfies and several psychological and social factors. Since adolescents and young adults make up a significant portion of the population and, if ignored, could eventually pose a major threat to public health, the correlations and association of these parameters with the various stages of development in the adolescent population serve as a foundation for additional research. This study revealed a positive correlation between perceived oral health and selfie-taking behaviour. However, no associations were found between selfie-taking behavior, narcissism, perceived oral health, mean DMFT score and mean plaque score. Further studies are required to assess selfie taking behaviour and oral health.

### Ethical and consent

Ethical clearance was obtained from the Institutional Ethics Committee of Manipal College of dental Sciences. Mangaluru [Protocol No: 18018 dated 10/03/18]. Informed Consent: Informed consent was obtained from all individual participants included in the study. Written informed consent was obtained from the study participants prior to the distribution of questionnaire.

## Data Availability

Figshare: Selfies, Narcissism and Oral health a cross - sectional study among Adolescents and young adults in Mangaluru - India. https://doi.org/10.6084/m9.figshare.26143195.v4 ^21^ This project contains the following underlying data:
• Dr Mithun Narrsisism and oral health.xlsx (Raw data from questionnaire responses) Data are available under the terms of the Creative Commons Zero “No rights reserved” data waiver (CC0 1.0 Public domain dedication). Figshare: Selfies, Narcissism and Oral health a cross - sectional study among Adolescents and young adults in Mangaluru - India. https://doi.org/10.6084/m9.figshare.26143195.v4 ^21^ This project contains the following underlying data:
•
Table 4: Binary logistic regression for Prediction of perceived oral health using narcissism, selfie behaviour and gender Data are available under the terms of the Creative Commons Zero “No rights reserved” data waiver (CC0 1.0 Public domain dedication). Figshare: *STROBE checklist for* Selfies, Narcissism and Oral health a cross - sectional study among Adolescents and young adults in Mangaluru - India. https://doi.org/10.6084/m9.figshare.26143195.v4 ^21^ Data are available under the terms of the Creative Commons Zero “No rights reserved” data waiver (CC0 1.0 Public domain dedication).
