# Supplementary material for: Selfies, narcissism and oral health a cross - sectional study among adolescents and young adults in Mangaluru - India
Source: F1000Res. 2024 Nov 11;13:809. Originally published 2024 Jul 17. [Version 3] doi: 10.12688/f1000research.153818.3 (PMC11568371; doi:10.12688/f1000research.153818.3)
Supplement: Peer review history

# Reviewer report: Reviewer response for version 3

**DOI:** 10.5256/f1000research.174112.r339418

**Published:** 2024-11-15

**Authors:** Venkitachalam Ramanarayanan

**Affiliations:** 1 Department of Public Health Dentistry, Amrita Vishwa Vidyapeetham, Kochi, Kerala, India

The authors have made changes as per suggestions. The article may be accepted for indexing

Is the work clearly and accurately presented and does it cite the current literature?

Partly

If applicable, is the statistical analysis and its interpretation appropriate?

Partly

Are all the source data underlying the results available to ensure full reproducibility?

Partly

Is the study design appropriate and is the work technically sound?

Partly

Are the conclusions drawn adequately supported by the results?

Partly

Are sufficient details of methods and analysis provided to allow replication by others?

No

Reviewer Expertise:

Dental Public Health, Dental Education, Knowledge synthesis

I confirm that I have read this submission and believe that I have an appropriate level of expertise to confirm that it is of an acceptable scientific standard.

# Reviewer report: Reviewer response for version 3

**DOI:** 10.5256/f1000research.174112.r339419

**Published:** 2024-11-15

**Authors:** Tarakant Bhagat, Ujwal Gautam

**Affiliations:** 1 BP Koirala Institute of Health Sciences, Dharan, Nepal; 2 Public Health Dentistry, B. P. Koirala Institute of Health Sciences, Dharan, Koshi, Nepal

The authors have tried to justify most of the queries. Hence, after reviewing the answers to the comments, we have given the final review.

Is the work clearly and accurately presented and does it cite the current literature?

Partly

If applicable, is the statistical analysis and its interpretation appropriate?

Partly

Are all the source data underlying the results available to ensure full reproducibility?

Yes

Is the study design appropriate and is the work technically sound?

Yes

Are the conclusions drawn adequately supported by the results?

Partly

Are sufficient details of methods and analysis provided to allow replication by others?

Partly

Reviewer Expertise:

Public health dentistry, epidemiology, oral health education,

We confirm that we have read this submission and believe that we have an appropriate level of expertise to confirm that it is of an acceptable scientific standard.

# Reviewer report: Reviewer response for version 2

**DOI:** 10.5256/f1000research.169733.r327803

**Published:** 2024-10-17

**Authors:** Pooja Latti

**Affiliations:** 1 Public Health Dentistry, Annoor Dental College and Hospital, Thodupuzha, India

**TITLE:** To add a comma after oral health

**ABSTRACT:** Background: Hence, a study was conducted with aim of the influence of selfie-taking behavior and narcissism on oral health – to revise the sentence. Methods: 1. To mention the sample population. 2. Oral health examination was performed to assess decay and plaque. To revise the sentence and mention it as Dental caries and to mention the methods used for recording dental caries and plaque status. Results: Requires grammatical corrections.

**BACKGROUND:**

 Adolescence is described as a transitional phase between childhood and adulthood, with a biological beginning (puberty) and a social ending. Adolescents are a susceptible group; they are the connect between young and old and are often the population that is routinely under scrutiny because of their diverse physical and psychological traits – *citation not mentioned for this paragraph.*

 The study was envisaged with the aim and objective of assessing correlations between perceived health, perceived oral health, selfie-taking behaviour, narcissism and oral health status using a standard questionnaire among a population of college students in Mangaluru, Karnataka **–** *The aim does not match with the title. The study does not aim to assess the correlation between health and selfie-taking behaviour, narcissism and hence to revise the above aim.*

 Rationale of the study is not mentioned with the relevant literature in relation to selfie taking behaviour and if it has a role on oral health.

**METHODS:**

 The students from the Department of Business Studies were the participants in the study – where were the students screened for oral health status? The sample was taken from how many institutions in Mangaluru?

 All the students are included – it can be mentioned as census survey.

 Eligibility criteria: Anxiety is common in people with narcissistic personalities, so these participants need to be included in the study. To justify with other reasons why this population as excluded in this study.

 Was the questionnaire validated?

 How was the dental caries and plaque status assessed?

**RESULTS:**

 Table 1, 2 and 5: To add Footnote to the tables and mention the significance of p value.

 Further evaluation between selfie behaviour and covariables revealed a positive significant correlation between gender and selfie behaviour – The gender distribution was unequal (60% males and 39% females) and hence the evaluation is not relevant in relation to gender context. Also, r value is not mentioned and just a sentence written saying there exists a positive significant correlation between gender and selfie behaviour.

 How was the selfie-taking behavior assessed?

**DISCUSSION:**

 There have been limited in the literature that have examined selfie behaviour in terms of perceived health, perceived oral health and dental status in a population – requires grammatical correction.

**LIMITATIONS:**

 The sample size was less and samples taken from only one institution.

 Could add recommendations.

**CONCLUSION:**

 Since adolescents and young adults make up a significant portion of the population and, if ignored, could eventually pose a major threat to public health, the correlations and association of these parameters with the various stages of development in the adolescent population serve as a foundation for additional research – This is a general sentence and does not reflect the results that are derived from this study.

 Conclusion to focus more on the results obtained in this study.

Is the work clearly and accurately presented and does it cite the current literature?

Partly

If applicable, is the statistical analysis and its interpretation appropriate?

Partly

Are all the source data underlying the results available to ensure full reproducibility?

Partly

Is the study design appropriate and is the work technically sound?

Partly

Are the conclusions drawn adequately supported by the results?

Partly

Are sufficient details of methods and analysis provided to allow replication by others?

Partly

Reviewer Expertise:

Dental Public Health, Bioethics, Preventive Dentistry, Epidemiology

I confirm that I have read this submission and believe that I have an appropriate level of expertise to confirm that it is of an acceptable scientific standard, however I have significant reservations, as outlined above.

# Reviewer report: Reviewer response for version 2

**DOI:** 10.5256/f1000research.169733.r327804

**Published:** 2024-10-09

**Authors:** Tarakant Bhagat, Ujwal Gautam

**Affiliations:** 1 BP Koirala Institute of Health Sciences, Dharan, Nepal; 2 Public Health Dentistry, B. P. Koirala Institute of Health Sciences, Dharan, Koshi, Nepal

**Background – last paragraph**

 Sufficient justification for conducting the study is lacking. Can add more on the rationale explaining the research gap this study is trying to address.

 How was the “Oral health status” defined for the study?

 Should elaborate on both subjective and objective interpretation measures of oral health employed in this study.

**Methods**

 Did the study consider entire duration of 2019-2021 or it was undertaken at some point during/after the implementation of oral health screening during the given years?

 Do you believe that the analysis using this sample size has adequate power to test the study's assumptions?

**Data collection procedure**

 What was the language employed while administering the questionnaires?

 The questionnaire to assess “selfie-taking behavior, perceived oral health, perceived general health” needs citation if it was taken from any source. If not, mention how the questionnaire was designed.

**Statistical analysis**

 Were assumptions of Normality assessed for Quantitative data?

 How were the qualitative data presented?

 Mention the different study outcomes and their corresponding study variables explicitly.

 Were assumptions for regression analysis examined?

 How did the investigators arrive at the model for regression? Elaborate on any underlying assumptions.

 Also, highlight the choice of forward stepwise technique.

**Results**

 Table 1: What is the basis for categorization of the responses to these variables for perceived general health, perceived oral health, DMFT index, and Plaque index?

 Table 2: Do the continuous variables satisfy the assumptions for the test?

**Limitations of the study**

 Should elaborate more on the generalizability of the study's findings.

Is the work clearly and accurately presented and does it cite the current literature?

Partly

If applicable, is the statistical analysis and its interpretation appropriate?

Partly

Are all the source data underlying the results available to ensure full reproducibility?

Yes

Is the study design appropriate and is the work technically sound?

Yes

Are the conclusions drawn adequately supported by the results?

Partly

Are sufficient details of methods and analysis provided to allow replication by others?

Partly

Reviewer Expertise:

Public health dentistry, epidemiology, oral health education,

We confirm that we have read this submission and believe that we have an appropriate level of expertise to confirm that it is of an acceptable scientific standard, however we have significant reservations, as outlined above.

# Reviewer report: Reviewer response for version 2

**DOI:** 10.5256/f1000research.169733.r327137

**Published:** 2024-10-09

**Authors:** Venkitachalam Ramanarayanan

**Affiliations:** 1 Department of Public Health Dentistry, Amrita Vishwa Vidyapeetham, Kochi, Kerala, India

Some comments have been revised. But several of them are not answered. It would be appreciated if a point by point rebuttal of the comments was provided. If any comment could not been revised or it doesn't warrant a revision, kindly provide a justification for the same.

Is the work clearly and accurately presented and does it cite the current literature?

Partly

If applicable, is the statistical analysis and its interpretation appropriate?

Partly

Are all the source data underlying the results available to ensure full reproducibility?

Partly

Is the study design appropriate and is the work technically sound?

Partly

Are the conclusions drawn adequately supported by the results?

Partly

Are sufficient details of methods and analysis provided to allow replication by others?

No

Reviewer Expertise:

Dental Public Health, Dental Education, Knowledge synthesis

I confirm that I have read this submission and believe that I have an appropriate level of expertise to confirm that it is of an acceptable scientific standard, however I have significant reservations, as outlined above.

# Reviewer report: Reviewer response for version 1

**DOI:** 10.5256/f1000research.168759.r304575

**Published:** 2024-07-25

**Authors:** Venkitachalam Ramanarayanan

**Affiliations:** 1 Department of Public Health Dentistry, Amrita Vishwa Vidyapeetham, Kochi, Kerala, India

Introduction: There is a disconnect between the paragraphs discussing about selfies/narcissism and perceived oral health. The rationale behind correlating the ideas of selfies, narcissism and oral health need to be elaborated further. 

 Methods: 

 "It is the educational centre in the state of Karnataka" - This statement can be removed as it is a general colloquial expression of the region. 

 Convenience was at the level of choosing the study population or sample? If all students were included from the target population, then you could categorize this as a census survey. 

 It would be worthy to add a note on why Department of Business Studies was chosen as the target population. 

 How was the questionnaire administered? Was it self-administered or investigator administered.

 Since narcissism is also linked to anxiety, what was the rationale of excluding them? They could have been retained and adjusted for in the analysis stage. Need an explanation for the exclusion.

 Placing data collection procedure section before statistical analysis section could ensure flow of ideas, especially with regard to the questionnaire used.  

 Results: 

 "96 of whom were males and 63 of whom were females" - I would recommend the used of percentages. 

 "The questionnaire included demographic information (age, sex, education, family monthly income, previous dental visits and medical history)" - The result section does not describe information on education, family monthly income and previous dental and medical history. 

 How was selfie behaviour present or absent defined ? Is there an accepted measure for this ? Information regarding this aspect could be added to the methodology section. 

 It is not clear how information on perceived oral and general health was collected? Was it a single question or multiple questions. I'm afraid that a self perceived general health questions would be a very vague concept. Kindly attach the questionnaire used for reference. The data file uploaded as a supplementary material does not contain expansion of the questions. 

 The types of narcissism - grandiose, vulnerable and total could be explained in detail in introduction or results section for a reader uninitiated in this concept. 

 'showed a statistically significant positive correlation' - what is observed based on the r values is a very weak positive correlation. R values needs to be interpreted based on its strength rather than direction and significance alone. 

 Table 3 - need to understand the definition of selfie taking behaviour to understand the table better. Mean value is of ??

 Binary logistic regression - how were the variables selected for the model. Is age a major factor as the population is more or less of same age category (young adults and adolescents)

 Discussion 

 Some information from first para of discussion on adolescents could be moved to introduction to give a context to study population. 

 'coastal' city - coastal word not relevant to the current context. 

 nearly no studies - "limited studies' could be a better terminology. 

 Was the self perceived oral health question focused on perceived esthetics? 

 "Participants with grandiose fantasy ^19^ have closely studied the effects of grandiose fantasy on personality traits and believe that people with grandiose fantasy tend to feel better for themselves." - why was this not observed with perceived general health? Any probable reason?

 "Egocentricity, grandiosity, arrogance, entitlement, envy ............................. " This paragraph is not linked to any of the study outcomes and seems out of place. Consider reframing it to suit the study objectives. 

 Conclusion

 The first part of conclusion could focus on the primary objective results. 

 "This study revealed a correlation between perceived oral health and selfie-taking behaviour." - What type of correlation -- weak/strong positive/negative? Otherwise this statement is incomplete. 

 OVERALL COMMENTS

 For the very reason that the study delves into unexplored areas, a strong and logical reasoning is warranted to link selfies with oral health. Inclusion of few variables like DMFT, SES etc does not seem to be connected to the objective. Further explanation regarding certain aspects is missing especially selfie behaviour definition. Certain statements also lack clarity and scientific language. Interpretation of certain results like correlation and regression needs to be improved.

Is the work clearly and accurately presented and does it cite the current literature?

Partly

If applicable, is the statistical analysis and its interpretation appropriate?

Partly

Are all the source data underlying the results available to ensure full reproducibility?

Partly

Is the study design appropriate and is the work technically sound?

Partly

Are the conclusions drawn adequately supported by the results?

Partly

Are sufficient details of methods and analysis provided to allow replication by others?

No

Reviewer Expertise:

Dental Public Health, Dental Education, Knowledge synthesis

I confirm that I have read this submission and believe that I have an appropriate level of expertise to confirm that it is of an acceptable scientific standard, however I have significant reservations, as outlined above.

# Response

**Published:** 2024-07-27

**Authors:** Mithun Pai

**Affiliations:** Manipal College Of dental Sciences , Mangalore, India

Respected Sir 

 We Thank you for the comments we have incorporated the necessary editions to the manuscript.
